# Autoimmune Thyroid Disease in Patients with Hypovitaminosis D in Department of Biochemistry of a Tertiary Care Centre: A Descriptive Cross-sectional Study

**DOI:** 10.31729/jnma.7493

**Published:** 2022-07-31

**Authors:** Ojaswee Sherchand, Apeksha Niraula, Bijaya Mishra, Manish Subedi, Robin Maskey

**Affiliations:** 1 Department of Biochemistry, Nepal Medical College and Teaching Hospital, Jorpati, Kathmandu, Nepal; 2 Department of Biochemistry, Institute of Medicine, Maharajgunj, Kathmandu, Nepal; 3 Department of Biochemistry, B.P. Koirala Institute of Health Sciences, Dharan, Sunsari, Nepal; 4 Department of Internal Medicine, B.P. Koirala Institute of Health Sciences, Dharan, Sunsari, Nepal; 5 Department of Internal Medicine, Mechi Zonal Hospital, Bhadrapur, Jhapa, Nepal

**Keywords:** *autoimmune diseases*, *prevalence*, *thyroid*, *vitamin D*

## Abstract

**Introduction:**

Autoimmune thyroid disease is characterised by the generation of autoantibodies against self-antigens such as thyroid peroxidase, thyroglobulin, and thyroid-stimulating hormone receptor. Recent studies have implicated the role of hypovitaminosis D to immune dysfunction, failure of self-tolerance and generation of autoantibodies. This study aimed to find out the prevalence of autoimmune thyroid disease among hypovitaminosis D patients in a tertiary care centre.

**Methods:**

A descriptive cross-sectional study was conducted among participants between the ages of 18 years to 65 years who visited the Department of Biochemistry of a tertiary care centre between the periods of July 2018 to December 2019. The study was initiated after receiving ethical approval from the Institutional Review Committee (Reference number: 42,8/074/075-IRC). Data was collected using a self-administered questionnaire followed by anthropometric measurement and blood collection. Thyroid hormone, thyroid peroxidase antibody and 25-hydroxy vitamin D were measured by chemiluminescence technique. Convenience sampling was used. Point estimate and 95% Confidence Interval were calculated.

**Results:**

Among 83 patients, 39 (46.98%) (42.32-51.63, 95% Confidence Interval) had autoimmune thyroid disease.

**Conclusions:**

The prevalence of autoimmune thyroid disease among patients with hypovitaminosis D was similar to studies conducted in comparable settings.

## INTRODUCTION

Autoimmune thyroid disease (AITDs) is one of the most prevalent organ specific autoimmune disorders.^1^ AITDs is marked by the production of autoantibodies; which may bind and stimulate thyroid stimulating hormone receptor resulting in Graves' disease or directed against thyroid antigens causing hypothyroidism (Hashimoto's thyroiditis).^2^ This occurs due to exposure of environmental adversaries upon a pre-existing genetic susceptibility.^3^ Recent studies have linked hypovitaminosis D with immune dysfunction and generation of autoantibodies.^4,5^

The immunomodulatory role of vitamin D in preventing autoimmune diseases by telorogenic dendritic cells induction has been widely explored.^5-9^ Studies shows lower levels of vitamin D can lead to autoimmune diseases as autoimmune thyroid.^10-12^ In contrast, a study assessing antithyroid peroxidase antibody (TPO-Ab) level in two categories of vitamin D; above and below 25 nmol/l of serum 25-hydroxy vitamin D [25(OH) D] found the prevalence of TPO-Ab was comparable between the groups.^13^

This study aimed to find out the prevalence of autoimmune thyroid disease among patients with hypovitaminosis D in a tertiary care centre.

## METHODS

A descriptive cross-sectional study was conducted to determine the prevalence of autoimmune thyroid disease among hypovitaminosis D patients visiting in the biochemistry laboratory of B.P. Koirala Institute of Health Sciences (BPKIHS). The study was initiated after receiving ethical approval from the Institutional Review Committee of BPKIHS (Reference number: 42,8/074/075-IRC). The patients of ages 18 years to 65 years who visited the laboratory for serum vitamin D and thyroid peroxidase test between the periods of July 2018 to December 2019 were enrolled in this study. The patients on vitamin D supplements, on thyroid medication or not providing written consent for participation were excluded from the study. Convenience sampling was done in this study.

Sample size was calculated using the following formula:


$$\begin{array}{ll} \text{n} & ={\text{Z}}^{2}\times \frac{\text{p}\times \text{q}}{{\text{e}}^{\text{2}}}\\ & =1.96^{2}\times \frac{0.50\times 0.50}{0.05^{2}}\\ & =385 \end{array}$$

Where,

n = minimum required sample size
Z = 1.96 at 95% Confidence Interval (CI)
p = prevalence taken as 50% for maximum sample size calculation
e = margin of error, 5%

Sample size was adjusted for finite population correction using the following formula: n


$$\begin{array}{ll} \text{n} & =\frac{\text{n}}{1+\frac{\text{(n}-1)}{\text{N}}}\\ & =\frac{\text{102}}{1+\frac{\text{(102}-1)}{\text{385}}}\\ & =81 \end{array}$$

Where,

n' = adjusted sample size for finite population
N = total number of para-pilots in Pokhara

However, 83 samples were taken in this study.

The participants were given self-administered questionnaires to obtain demographic information, history of comorbid conditions (type 1 diabetes mellitus, hypertension, clinically diagnosed mental illness), exposure to radiation, and smoking. Data collection was followed by anthropometric measurements of height and weight using standard protocol. Body mass index (BMI) was calculated and classified as normal weight (18.5-22.9 kg/m^2^), overweight (≥23.0-24.99 kg/m^2^), and obese (≥25 kg/m^2^).^14^

Venous blood samples were collected and serum was used to measure 25(OH)D, thyroid hormones; triiodothyronine (fT3), tetraiodothyronine (fT4) and thyroid stimulating hormone (TSH) and TPO-Ab. Tests were performed as per standard protocol in fully automated Maglumi 1000 analyzer (SNIBE Co, Ltd, China) using chemiluminescence immunoassay (CLIA) technique.

The serum 25(OH)D below 30 ng/ml as hypovitaminosis D.^15^ In Hypovitaminosis D serum 25(OH)D between 2920 ng/ml is vitamin D insufficient and <20 ng/ml is vitamin D deficient. The laboratory reference interval of thyroid hormone was as follows; fT3 (1.21-4.18 pg/ml), fT4 (8.9-17.2 pg/ml), TSH (0.3-4.5 μIU/ml). Values above or below the reference interval were used to establish hypo or hyperthyroidism; collectively classified as thyroid dysfunction while those having normal reference values were classified as euthyroid.

TPO-Ab in serum was measured by Sandwich immunoluminometric assay using Maglumi Anti-TPO (CLIA) kits. The upper reference limit for TP0-Ab>30 IU/ml was used as recommended by the manufacturer. Thus, autoimmune thyroid disease (AITD) was defined as those cases clinically diagnosed by a physician and having TPO antibody (TP0-Ab)>30 IU/ml irrespective of thyroid hormone status.^16^ Those having TPO-Ab≤30 were categorised as non-AITD.

Data were analysed using IBM SPSS Statistics 11.5. Point estimate and 95% CI were calculated.

## RESULTS

Out of 83 patients with hypovitaminosis D, 39 (46.98%) had AITD (42.32-51.63, 95% CI). Majority 29 (74.35%) of the patients had deranged thyroid function tests and 26 (66.67%) of the patients were vitamin D deficient.

**Table 1:** Features of patients with AITD (n = 39).

Features	n (%)
**Thyroid function test**	
Abnormal	29 (74.36)
Euthyroid	10 (25.64)
Obese	24 (61.54)
Non-smokers	37 (94.87)
No radiation exposure within past 6 months	35 (89.74)
Comorbidity	2 (5.13)
**Hypovitaminosis D**	
Vitamin D deficient	26 (66.67)
Vitamin D insufficient	13 (33.33)

The mean of age of the patients with AITD was 35.60±9.30 years and the mean BMI was 26.36±3.78 kg/m^2^. Also, the mean level of the 25(OH)D was 19.16±5.60 ng/ml. The levels of the thyroid tests are given below (Table 2).

**Table 2:** Thyroid tests of the patients with AITD (n= 39).

Tests	Mean±SD
Free T3 (pg/ml)	2.64±0.47
Free T4 (pg/ml)	11.57±2.80

In the patients with AITD, the median TPO-Ab was found to be 371.8 with interquartile range (IQR): 291.50-609.75 (Table 3).

**Table 3:** Thyroid tests of the patients with AITD (n = 39).

Tests	Median (IQR)
TPO-Ab (IU/ml)	371.8 (291.50-609.75)
TSH (IU/ml)	7 (3-8)

A total of 28 (33.73%) patients were Indo-Aryan, 33 (39.75%) were women, 36 (43.37%) were Hindus and 30 (36.14%) were from the Sunsari region (Table 4).

**Table 4:** Sociodemographic variables in patients with AITD (n= 39).

Variables	n (%)
**Sex**	
Females	33 (84.61)
Males	6 (15.38)
**Age**	
≤45 years	35 (89.74)
>45 years	4 (10.26)
**Ethnolinguistic group**	
Indo-Aryan	28 (71.79)
Tibeto-Burman	11 (28.20)
**Religion**	
Hindus	36 (92.30)
Others	3 (7.69)
**Location**	
Sunsari	30 (76.92)
Others	9 (23.07)

## DISCUSSION

Our study consisted of patients suffering from hypovitaminosis D amongst which 39 (46.98%) had autoimmune thyroid disease. The prevalence of AITD was higher among vitamin D deficient patients.

Vitamin D can bind and modulate antigen presenting cells as dendritic cells, monocyte, macrophages as well as T and B cell functions and hence crucial for proper immune functions.^17^ Low levels of vitamin D can dampen the immunity and lead to failure of selftolerance resulting in autoimmunity.^10^ We found most AITD patients were in mid-thirties. Higher circulating sex hormones during reproductive age can moderate the mechanisms of thyroid autoimmunity.^18^

Majority of the study participants were women; hence it was difficult to make gender comparisons in the prevalence of AITD. However, studies have shown a higher prevalence of AITD in women.^19,20^ Hormonal changes throughout woman's life as menarche, pregnancy, and menopause may breed the ground for autoimmune disease.^20^ A study conducted found premenopausal women with lower vitamin D had significantly higher prevalence of AITD than postmenopausal women or male.^11^

Along with AITD, body mass index was also measured. Body mass index gives a measure of body fat per height, which is categorized as underweight, normal, overweight and obese. In our study, we categorized basal metabolic index for Asia-pacific body type.^14^ A higher prevalence of AITD was found in obese people. In the recent years obesity has been recognized as an endocrine organ that releases pro-inflammatory cytokines called adipokines which leads failure of selftolerance and development of autoimmunity.^21^

Thyroid hormone tests showed most participants had normal serum T3 and T4 irrespective of their TPO-Ab status. However, the mean TSH was higher than the normal reference interval in AITD group. Thyroid hormones levels seems to vary depending on the stage and type of AITD, with hypothyroidism occurring in Hashimoto thyroiditis and hyperthyroidism in Graves' disease.^22^ However, in our study we did not further classify AITD into Hashimoto or Graves' disease, nor did we know the stage of AITD hence the reason for elevated TSH in our case cannot be subjected to this cause.

There were some limitations in our study. Due to the descriptive nature of our study, we could not establish causality. Due to small sample size, we did not classify AITD; hence the variable effect of low vitamin D in the prevalence of different types of AITD could not be made.

## CONCLUSIONS

The prevalence of autoimmune thyroid disease among patients with hypovitaminosis D was similar studies conducted in comparable settings. Further large-scale prospective study needs to be conducted to establish causal effect of hypovitaminosis D in the pathogenesis of AITD.
